# Human translatability of the GAN diet-induced obese mouse model of non-alcoholic steatohepatitis

**DOI:** 10.1186/s12876-020-01356-2

**Published:** 2020-07-06

**Authors:** Henrik H. Hansen, Helene M. Ægidius, Denise Oró, Simon S. Evers, Sara Heebøll, Peter Lykke Eriksen, Karen Louise Thomsen, Anja Bengtsson, Sanne S. Veidal, Michel Feigh, Malte P. Suppli, Filip K. Knop, Henning Grønbæk, Diego Miranda, James L. Trevaskis, Niels Vrang, Jacob Jelsing, Kristoffer T. G. Rigbolt

**Affiliations:** 1 Gubra, DK-2970 Hørsholm, Denmark; 2 Department of Hepatology and Gastroenterology, Aarhus University Hospital, Aarhus, Denmark; 3 Center for Clinical Metabolic Research, Gentofte Hospital, University of Copenhagen, Hellerup, Denmark; 4 Department of Clinical Medicine, Faculty of Health and Medical Sciences, University of Copenhagen, Copenhagen, Denmark; 5 Novo Nordisk Foundation Center for Basic Metabolic Research, Faculty of Health and Medical Sciences, University of Copenhagen, Copenhagen, Denmark; 6 Gilead Sciences, Foster City, CA USA

**Keywords:** Non-alcoholic steatohepatitis, Mouse model, Diet-induced obesity, Translatability, Histopathology, Histomorphometry, Liver transcriptome, Glucose tolerance

## Abstract

**Background:**

Animal models of non-alcoholic steatohepatitis (NASH) are important tools in preclinical research and drug discovery. Gubra-Amylin NASH (GAN) diet-induced obese (DIO) mice represent a model of fibrosing NASH. The present study directly assessed the clinical translatability of the model by head-to-head comparison of liver biopsy histological and transcriptome changes in GAN DIO-NASH mouse and human NASH patients.

**Methods:**

C57Bl/6 J mice were fed chow or the GAN diet rich in saturated fat (40%), fructose (22%) and cholesterol (2%) for ≥38 weeks. Metabolic parameters as well as plasma and liver biomarkers were assessed. Liver biopsy histology and transcriptome signatures were compared to samples from human lean individuals and patients diagnosed with NASH.

**Results:**

Liver lesions in GAN DIO-NASH mice showed similar morphological characteristics compared to the NASH patient validation set, including macrosteatosis, lobular inflammation, hepatocyte ballooning degeneration and periportal/perisinusoidal fibrosis. Histomorphometric analysis indicated comparable increases in markers of hepatic lipid accumulation, inflammation and collagen deposition in GAN DIO-NASH mice and NASH patient samples. Liver biopsies from GAN DIO-NASH mice and NASH patients showed comparable dynamics in several gene expression pathways involved in NASH pathogenesis. Consistent with the clinical features of NASH, GAN DIO-NASH mice demonstrated key components of the metabolic syndrome, including obesity and impaired glucose tolerance.

**Conclusions:**

The GAN DIO-NASH mouse model demonstrates good clinical translatability with respect to the histopathological, transcriptional and metabolic aspects of the human disease, highlighting the suitability of the GAN DIO-NASH mouse model for identifying therapeutic targets and characterizing novel drug therapies for NASH.

## Background

Non-alcoholic fatty liver disease (NAFLD) comprises a continuum of liver lesions ranging from simple steatosis to non-alcoholic steatohepatitis (NASH) which, in addition to steatosis, is characterized by lobular inflammation and hepatocellular ballooning degeneration [1]. NASH has emerged as a major challenge for public health because of its increasing prevalence worldwide, difficulties in diagnosis, risk of severe complications and lack of effective therapies. The presence of the metabolic syndrome, notably obesity and type 2 diabetes, is the strongest predisposing factor for development and progression of NAFLD [2]. While simple steatosis usually has a benign course, patients with NASH and fibrosis carry an increased risk of liver-related complications, including cirrhosis, hepatocellular carcinoma and end-stage liver disease. As a consequence, NASH is expected soon to become the leading indication for liver transplantation [3].

Given the lack of effective therapies for NASH, there is a need to establish animal models that better predict clinical responses. The increased understanding of various pathogenic drivers acting together (‘multiple hits’) to trigger the onset and progression of NASH has played an important role in the development of animal models of NASH with reproducible and robust metabolic and liver histopathological changes [4, 5]. Although no single rodent model of NAFLD recapitulates the full spectrum of the human condition, the clinical and histopathological hallmarks of NASH are largely reproducible in diet-induced obese (DIO) mice fed ‘Western’ high-fat diets specifically modified to enhance NASH pathology and promote liver fibrosis [5, 6], making these models highly relevant in preclinical drug discovery [7]. In particular, high intake of saturated fat, trans-fats, fructose and cholesterol is associated with increased risk of NAFLD/NASH [8–10]. A prototypic example of an experimental NASH-promoting diet rich in these components is the ALIOS (American lifestyle-induced obesity syndrome) diet initially described by Tetri and colleagues [11]. We have further refined and validated this diet formula, termed the Amylin liver NASH (AMLN) diet [12], for reliably inducing metabolic, biochemical and liver-biopsy confirmed histological changes recapitulating hallmarks of NASH in C57BL/6 J (AMLN DIO-NASH) and leptin-deficient *ob/ob* (AMLN *ob/ob*-NASH) mice. The two AMLN models have been increasingly used in preclinical drug development for NASH [12–20]. However, a recent regulatory ban on trans-fats as food additive [21] has prompted the development of a compatible Western diet capable of promoting metabolic and liver histopathological changes similar to the AMLN diet. We have recently reported maintained fibrotic NASH histopathology in C57BL/6 J (GAN DIO-NASH) and *ob/ob* (GAN *ob/ob*) mice fed a modified, trans-fat free AMLN diet termed the Gubra Amylin NASH (GAN) diet [22].

The present study aimed to further validate the clinical translatability of GAN diet-induced liver lesions by comparing liver biopsy histopathological and transcriptome characteristics in GAN DIO-NASH mice and human NASH patients.

## Methods

### Human subjects

Healthy normal-weight individuals (*n* = 14, body mass index 18.5–25 kg/m2) were recruited at Center for Clinical Metabolic Research, Gentofte Hospital, University of Copenhagen (Hellerup, Denmark). Normal-weight individuals were not suffering from or showing symptoms of liver disease or other illnesses. NASH patients (*n* = 16) were diagnosed and recruited at the Department of Hepatology and Gastroenterology, Aarhus University Hospital (Aarhus, Denmark) as described in detail previously [23–25]. The HOMA-IR (homeostasis model assessment of insulin resistance) index was calculated as [fasting plasma insulin (mU/L) x fasting blood glucose (mmol/L)/22.5] [26], based on fasting blood glucose and plasma insulin concentrations reported previously [23–25]. All included participants underwent liver biopsy for histological evaluation. Percutaneous liver biopsy was performed under ultrasound guidance. The liver biopsy was divided into a > 10 mm sample (> 10 portal tracks) and fixed in phosphate-buffered formalin for histological evaluation. The remaining sample material was placed in RNAlater (ThermoFisher Scientific, Waltham, MA) or snap-frozen in liquid nitrogen and stored at − 80°C until later processing. Diagnosis of NASH was based on confirmation of lobular inflammation and hepatocyte ballooning in addition to steatosis, see below.

### Animals

The Danish Animal Experiments Inspectorate approved all experiments which were conducted using internationally accepted principles for the use of laboratory animals (license #2013-15-2934-00784). C57BL/6 J mice (5 weeks old) were from Janvier Labs (Le Genest Saint Isle, France) and housed in a controlled environment (12 h light/dark cycle, lights on at 3 AM, 21 ± 2 °C, humidity 50 ± 10%). Each animal was identified by an implantable subcutaneous microchip (PetID Microchip, E-vet, Haderslev, Denmark). Mice had ad libitum access to tap water and chow (3.22 kcal/g, Altromin 1324, Brogaarden, Hoersholm, Denmark) or Gubra Amylin NASH diet [GAN diet, 4.49 kcal/g, 40 kcal-% fat (of these 46% saturated fatty acids by weight), 22% fructose, 10% sucrose, 2% cholesterol; D09100310, Research Diets]. Mice were fed chow or GAN diet for 38–44 weeks. Animals were terminated by cardiac puncture under isoflurane anesthesia.

### Histopathology scoring

Semiquantitative histopathological scoring was performed on HE- and PSR-stained human (healthy normal-weight individuals, *n* = 14; NASH patients, *n* = 16) and mouse (chow-fed mice, *n* = 10; GAN DIO-NASH mice, *n* = 25) liver samples in a blinded manner by experienced hepato-histopathologists using the NAFLD Activity Score (NAS) and fibrosis staging system according to the Brunt criteria, modified by Kleiner et al. [27]. The NAS score was defined as the unweighted sum of the scores for steatosis (0–3), lobular inflammation (0–3), and ballooning (0–2), thus ranging from 0 to 8. Liver fibrosis was scored based on a five-point scale, i.e. absence of fibrosis (stage 0), perisinusoidal or portal fibrosis (stage 1), perisinusoidal and portal/periportal fibrosis (stage 2), septal or bridging fibrosis (stage 3), cirrhosis (and stage 4).

### Liver histomorphometry

Human liver biopsies (healthy normal-weight individuals, *n* = 14; NASH patients, *n* = 11) and mouse lobular samples (chow-fed mice, *n* = 10; GAN DIO-NASH mice, *n* = 25) were fixed overnight in 4% paraformaldehyde, paraffin-embedded and sectioned (3 μm thickness). Sections were stained with hematoxylin-eosin (HE), anti-galectin-3 (cat. 125,402, Biolegend, San Diego, CA) or picro-Sirius Red (PSR, Sigma-Aldrich, Broendby, Denmark) using standard procedures [28, 29]. The fractional (%) area of liver lipid (HE-staining), inflammation (galectin-3) and fibrosis (PSR) was expressed relative to total sectional area.

### Liver RNA sequencing analysis

Liver transcriptome analysis was performed by RNA sequencing on RNA extracts from human liver biopsies (healthy normal-weight individuals, *n* = 14; NASH patients, *n* = 16) and mouse lobular liver samples (chow-fed mice, *n* = 10; GAN DIO-NASH mice, *n* = 25), as described in detail elsewhere [25, 28]. The RNA quantity was measured using Qubit® (Thermo Scientific, Eugene, OR). The RNA quality was determined using a bioanalyzer with RNA 6000 Nano kit (Agilent, Waldbronn, Germany). All samples were confirmed to have high-quality RNA (RNA integrity number ≥ 7.5). RNA sequence libraries were prepared with NeoPrep (Illumina, San Diego, CA) using the Illumina TruSeq stranded mRNA Library kit and sequenced on the NextSeq 500 System (Illumina, San Diego, CA) with NSQ 500 hi-Output KT v2 (75 CYS, Illumina, San Diego, CA). Reads were aligned to the GRCh38.p10 Ensembl human genome or GRCm38 v84 Ensembl *Mus musculus* genome using STAR v.2.5.2a with default parameters. For both human and mouse samples, a lower detection limit for gene expression was defined based on raw mapped read counts (RPKM = 0.1). The R package DESeq2 v.1.18.1 [30] was used for differential expression analysis. *P*-values were adjusted using the Benjamini-Hochberg method, and a cut-off of 0.05 (5% False Discovery Rate, FDR) was applied. Candidate NAFLD- and fibrosis-associated pathways were used to annotate genes involved in disease progression (Table S1). A gene set analysis was conducted with the R package PIANO version 1.18.1 using the Stouffer method, and *p*-values were corrected for multiple testing using the Benjamini-Hochberg method (FDR < 0.05). The Reactome pathway database was retrieved and used as gene annotation for global transcriptional changes (*p* < 0.05).

### Glucose tolerance test

An intraperitoneal glucose tolerance test (ipGTT) was performed in chow-fed mice (*n* = 10) and GAN DIO-NASH mice (*n* = 15) one week prior to termination. Animals were fasted for 4 h whereupon an intraperitoneal glucose bolus (2 g glucose/kg, 10 mL/kg; Fresenius Kabi, Uppsala, Sweden) was administrated at t = 0. A baseline blood sample (− 60 min) and successive blood samples during the ipGTT (0–180 min) were collected from the tail vein (20 μl per sample) for measuring blood glucose. Glucose area under the curve (AUC, 0–180 min) was determined.

### Plasma and liver biochemistry

Terminal 4 h fasting whole blood was sampled from chow-fed mice and GAN DIO-NASH mice, kept on ice and centrifuged (5 min, 4 °C, 6000 x g) to generate EDTA-stabilized plasma assayed for insulin, leptin, free fatty acids, triglycerides (TG), total cholesterol (TC), alanine aminotransferase (ALT) and aspartate aminotransferase (AST) as described previously [14, 28]. HOMA-IR index was calculated using similar procedure as for clinical samples (see above). Plasma markers for fibrosis were determined utilizing recommended sample dilutions and standard curve concentrations for total cytokeratin 18 (CK-18, #CSB-E17158m, Cusabio, Houston, TX), matrix metalloproteinase-9 (MMP-9, #AL519C, PerkinElmer, Waltham, MA) and tissue inhibitor of metalloproteinase-1 (TIMP-1, #MTM100, R&D Systems, Minneapolis, MN). Liver lobular samples from chow-fed mice and GAN DIO-NASH mice were analyzed for TG, TC and hydroxyprolin (HP) as reported previously [14, 28]. A multiplex assay was applied for cytokine analysis (TNF-α, interferon-γ, IL-1β, IL-2, IL-4, IL-5, IL-6, IL-10, KC/GRO (CXCL1), IL12p70) in mouse plasma samples and liver lobular homogenates according to the manufacturer’s protocol (#K15048G, Meso Scale Diagnostics, Rockville, MD). A separate kit was used for analysis of plasma and liver concentrations of mouse MCP-1 (CCL-2, #K152NND, Meso Scale Diagnostics, Rockville, MD).

### Statistical analysis

All results are shown as mean ± standard error of mean (S.E.M.). Except from RNA sequencing, an unpaired t-test (quantitative histology, plasma/liver biochemistry and plasma cytokine data) or a two-way ANOVA with Dunnett’s multiple comparison test (ipGTT data) was applied. A *p*-value < 0.05 was considered statistically significant.

## Results

### Comparable liver histopathological hallmarks GAN DIO-NASH mice and NASH patients

**Fig. 1:**
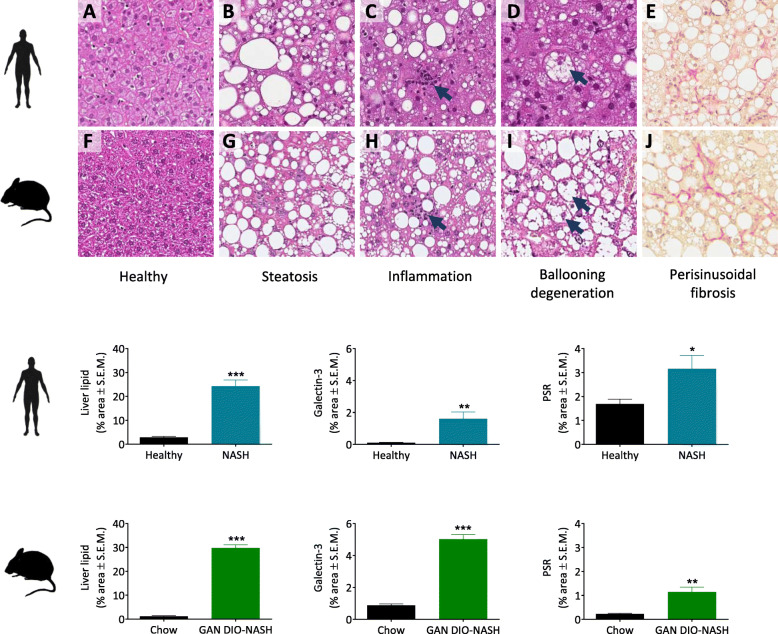
Similar liver histopathological hallmarks in human NASH patients and GAN DIO-NASH mice. Upper panels: Representative photomicrographs of liver sections from human healthy normal-weight individual (**a**), NASH patient (**b**-**e**), chow-fed mouse (**f**) and GAN DIO-NASH mouse (**g**-**j**) stained with hematoxylin-eosin (HE, for evaluation of steatosis, inflammation and hepatocyte ballooning; **a**-**d**, **f**-**i**) or picro-Sirius Red (PSR, for evaluation of fibrosis; **e**, **j**). Arrows indicate inflammatory foci and ballooning hepatocytes, respectively. Lower panels: Histomorphometric quantitative assessment of liver lipid accumulation (HE staining), inflammation (galectin-3 immunostaining) and fibrosis (PSR staining) in human and mouse liver biopsy sections. *p < 0.05, **p < 0.01, ***p < 0.001 vs. corresponding control group, unpaired t-test

In order to assess the translational relevance of the mouse model, liver histopathological changes in GAN DIO-NASH mice were compared to liver biopsy samples from human NASH patients. GAN DIO-NASH mice showed similar morphological appearance of steatosis, inflammatory foci and hepatocyte ballooning as compared to human NASH, see representative histological stainings in Fig. 1. Hepatocyte lipid accumulation was distributed throughout the liver lobule in GAN DIO-NASH mice, appearing mainly as macrovesicular steatosis (single large intracytoplasmic fat droplet or smaller well-defined droplets), displacing the nucleus to the cell periphery. Lobular inflammation in GAN DIO-NASH mice was characterized by small foci of inflammatory cells showing mainly macrophage and lymphocyte morphology. In both GAN DIO-NASH mice and NASH patients, hepatocyte ballooning degeneration was observed in the centrilobular zone 3 appearing as swollen hepatocytes with pale and rarefied cytoplasm. Perisinusoidal collagen fiber distribution in GAN DIO-NASH mice was similar to that observed in liver biopsy samples from NASH patients. Mild fibrosis (stage F1) presented as periportal or perisinusoidal fibrosis in GAN DIO-NASH mice while perisinusoidal fibrosis was more prevalent in the NASH patient sample set. Moderate-stage fibrosis (stage F2) with portal and central zonation was similar in GAN DIO-NASH mice and NASH patients.

Individual histopathological scores in mouse and human liver biopsies are indicated in Table 1. Healthy normal-weight human individuals had normal-range liver histology. NASH patients showed moderate-severe steatosis (grade 2–3), mild lobular inflammation (grade 1) and mild to prominent hepatocyte ballooning (grade 1–2). 13 out of 16 NASH patients were diagnosed with fibrosis ranging from mild to moderate stage (F1-F2). In comparison to normal liver histology in chow-fed control mice, all GAN DIO-NASH mice showed severe steatosis (grade 3) with varying severity of lobular inflammation. When present, hepatocyte ballooning degeneration was mild (grade 1). 22 out of 25 GAN DIO-NASH mice developed liver fibrosis, nearly all presenting mild to moderate stage fibrosis (F1-F2). The relatively similar disease severity in NASH patients and GAN DIO-NASH mice was reflected by comparable NAFLD Activity Scores (NASH patients, NAS 5–6; GAN DIO-NASH mice, NAS 4–7), see Table 2. HOMA-IR was significantly increased in NASH patients (7.6 ± 1.0, *p* < 0.0001, *n* = 16) compared to healthy normal-weight individuals (1.3 ± 0.2, *n* = 14).

**Table 1:** Comparison of liver biopsy histopathology scores in human NASH patients and GAN DIO-NASH mice

	Group	***n***	Steatosis score				Inflammation score				Ballooning score			Fibrosis stage				
			0	1	2	3	0	1	2	3	0	1	2	0	1	2	3	4
**Human subjects**	Healthy normal-weight	14	13	1	–	–	14	–	–		14	–	–	14	–	–	–	–
	NASH	16	–	–	2	14	–	16	–		–	10	6	3	12	1	–	–
**C57BL/6 J mice**	Chow-fed lean mice	10	10	–	–	–	10	–	–		10	–	–	10	–	–	–	–
	GAN DIO-NASH mice	25	–	–	–	25	–	6	13	6	18	7	–	3	10	11	1	–

Histopathology scores in liver biopsy samples from normal controls, human NASH patients and GAN DIO-NASH mice. Histopathology was scored according to the criteria outlined by the NASH-Clinical Research Network [27].

**Table 2:** Comparison of composite NAFLD Activity Score (NAS) in human NASH patients and GAN DIO-NASH mice

			NAS								
	Group	***n***	0	1	2	3	4	5	6	7	8
**Human subjects**	Healthy normal-weight	14	13	1	–	–	–	–	–	–	–
	NASH	16	–	–	–	–	–	12	4	–	–
**C57BL/6 J mice**	Chow-fed lean mice	10	10	–	–	–	–	–	–	–	–
	GAN DIO-NASH mice	25	–	–	–	–	6	7	11	1	–

Composite NAFLD Activity Score (NAS) in liver biopsy samples from normal controls, human NASH patients and GAN DIO-NASH mice. Histopathology was scored according to the criteria outlined by the NASH-Clinical Research Network [27]

### Comparable quantitative liver histopathological changes in GAN DIO-NASH mice and NASH patients

Quantitative levels of liver fat (HE staining), inflammatory cell infiltration (galectin-3 IHC) and collagen deposition (PSR staining) were compared in human and mouse liver biopsies (Fig. 1). NASH patients (*n* = 11) demonstrated significant increases in the fractional (%) area of liver fat (8-fold increase; 24.2 ± 2.6 vs. 2.9 ± 0.4%, *p* < 0.0001), galectin-3 (16-fold increase; 1.6 ± 0.4 vs 0.1 ± 0.02, *p* = 0.0016) and PSR (2-fold increase; 3.2 ± 0.6 vs. 1.7 ± 0.2, *p* < 0.0122) as compared to normal-weight healthy individuals (*n* = 12–14). Correspondingly, GAN DIO-NASH mice (*n* = 25) showed significant increases in % area of liver fat (25-fold increase; 29.8 ± 1.3 vs. 1.2 ± 0.2%, *p* < 0.0001), galectin-3 (6-fold increase; 5.0 ± 0.3 vs 0.9 ± 0.1%, p < 0.0001) and PSR (5-fold increase; 1.1 ± 0.2 vs. 0.2 ± 0.02%, *p* = 0.0064) as compared to chow-fed lean mice (*n* = 10).

### Overlapping hepatic transcriptome profiles in GAN DIO-NASH mice and NASH patients

**Fig. 2:**
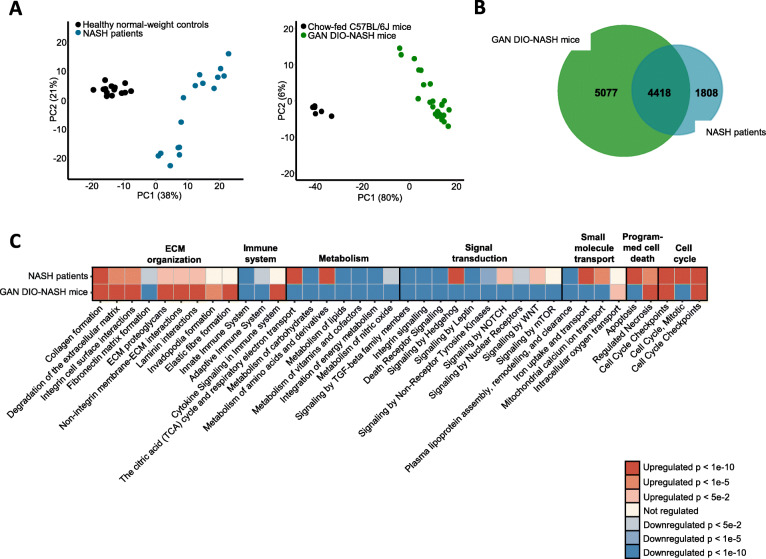
Liver transcriptome changes in human NASH patients and GAN DIO-NASH mice. **a** Principal component analysis (PCA) of samples based on top 500 most variable gene expression levels. **b** Venn diagram depicting shared and separate differentially expressed genes (DEGs; false discovery rate < 0.05) in NASH patients and GAN DIO-NASH mice. **c** Comparison of significantly regulated disease-associated Reactome signalling pathways in NASH patients (*n* = 16) and GAN-DIO NASH mice (*n* = 25). Reactome signalling pathways are grouped according to biological pathway. Color gradients indicate significantly upregulated (red color) and downregulated (blue color) pathways compared to corresponding control group (chow-fed mice, *n* = 10; healthy normal-weight human individuals, *n* = 14)

**Fig. 3:**
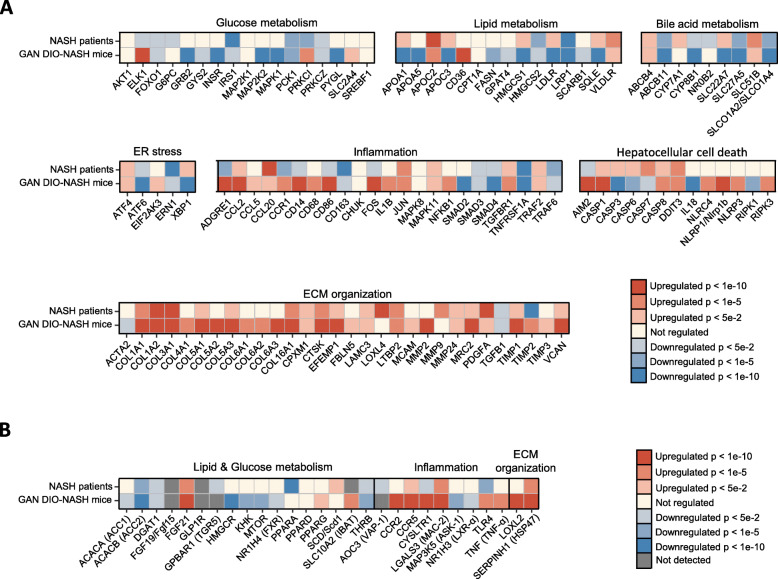
Regulation of disease-associated candidate genes in NASH patients and GAN DIO-NASH mice. **a** Disease-associated hepatic candidate genes, **b** Selected hepatic genes representing various drug targets for NASH. Color gradients indicate significantly upregulated (red color) and downregulated (blue color) gene expression in NASH patients (*n* = 16) and GAN DIO-NASH mice (*n* = 25) compared to corresponding control group (healthy normal-weight human individuals, n = 14; chow-fed mice, *n* = 10). Candidate genes are listed in Table S1

**Fig. 4:**
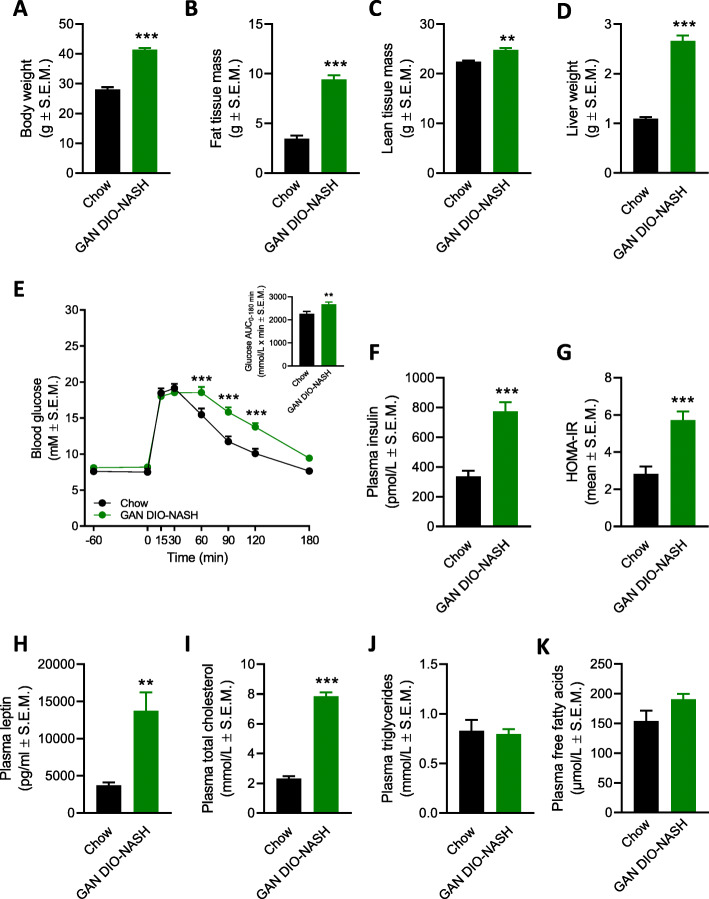
GAN DIO-NASH mice develop characteristics of the metabolic syndrome. Terminal body weight (**a**), whole-body fat mass (**b**), whole-body lean mass (**c**), liver weight (**d**). **p < 0.01, ***p < 0.01 (unpaired t-test; chow-fed mice, *n* = 6–10; GAN DIO-NASH mice, n = 14–16). An intraperitoneal glucose tolerance test (ipGTT) was performed one week before termination. (**e**) Glucose excursion curves (− 60 to 180 min). ***p < 0.001 (repeated-measure two-way ANOVA; chow-fed mice, *n* = 10; GAN DIO-NASH mice, *n* = 15). Insert, AUC-glucose levels (0–180 min). **p < 0.01 (unpaired t-test). Terminal plasma insulin (**f**) and HOMA-IR (**g**). ***p < 0.01 (unpaired t-test; chow-fed mice, *n* = 10; GAN DIO-NASH mice, n = 15). Terminal plasma leptin (**h**), total cholesterol (**i**), triglycerides (**j**) and free fatty acids (**k**). **p < 0.01, ***p < 0.01 (unpaired t-test; chow-fed mice, *n* = 5–10; GAN DIO-NASH mice, *n* = 8–15)

To compare global gene expression profiles in mouse and human liver biopsy samples, a principal component analysis (PCA) was performed (Fig. 2a). The primary PCA, accounting for the major variability in the data set, indicated that liver transcriptome profiles in healthy normal-weight human controls were clearly separated from the transcriptome profiles in NASH patient samples. This also applied to chow-fed controls vs. GAN DIO-NASH mice. GAN DIO-NASH mice demonstrated relatively homogenous gene expression changes as indicated by close clustering of individual transcriptome signatures. An extensive number of differentially expressed genes (DEGs) were determined in liver biopsies from GAN DIO-NASH mice (*n* = 9495) and NASH patients (*n* = 6226) with a significant overlap in DEGs (*n* = 4418), see Fig. 4b. A gene set enrichment analysis revealed significant perturbations in Reactome top level signaling pathways in GAN DIO-NASH mouse and NASH patient samples. To a large extent, similar directional shifts in top canonical NASH-associated pathways were detected in GAN DIO-NASH mice and NASH patients. Reflecting key metabolic and histopathological features of NASH, most consistent pathway perturbations in GAN DIO-NASH mice and NASH patients were associated with nutrient metabolism, immune function and extracellular matrix (ECM) organization (Fig. 2c). To obtain further resolution of hepatic transcriptome regulations in GAN DIO-NASH mice and NASH patients, RNA sequencing data were probed for candidate genes linked to NASH pathology and fibrosis (Fig. 3, Table S1). Compared to NASH patients, GAN DIO-NASH mice showed more widespread regulations within the seven defined gene categories. Changes in gene transcriptional programs pointed to deficient glucose metabolism, impaired lipid and bile acid handling, immunomodulation, endoplasmic reticulum (ER) stress, hepatocellular injury and enhanced ECM remodeling activity (Fig. 3a). A subset of candidate genes encode protein targets exploited for the treatment of NASH [7, 31, 32]. These 28 genes were grouped into three major categories according to major function, i.e. Lipid & Glucose metabolism, Inflammation and ECM organization (Fig. 3b). GAN DIO-NASH mice and NASH patients demonstrated significantly regulated genes within each category. Twenty-five out of twenty-eight drug target-associated genes were significantly regulated in GAN DIO-NASH mice, including downregulation of *ACACA/ACC1, ACACB/ACC2, DGAT1, HMGCR, KHK, MAP 3 K5/ASK1, MTOR, NR1H3/LXR-α, NR1H4/FXR, THRB* and upregulation of *CCR2, CCR5, CYSLTR1, FGF21, LGALS3/MAC-2, LOXL2, PPARG, SLC10A2/IBAT, SERPINH1/HSP47, TLR4, TNF/TNF-α*. In NASH patient liver biopsies, similar gene expression signatures were observed for *ACACB/ACC2, DGAT1, THRB* (downregulated) and *CCR5, FGF21, LGALS3/MAC-2, SERPINH1/HSP47* (upregulated). In addition, NASH patients showed downregulation of *PPARA, AOC3/VAP-1, CYSLTR1, GPBAR1/TGR5, TLR4* and upregulation of *SCD*. mRNA expression of *FGF15/19* and *GLP1R* was not detected in mouse and human liver samples, being in agreement with previous reports [33–35]. *AOC3/VAP-1* and *GPBAR1/TGR5* expression were below detection limit in liver samples from chow-fed and GAN DIO-NASH mice which is likely ascribed to selective expression in non-parenchymal liver cell populations [36, 37]. In line with previous reports [38, 39], *SLC10A2/IBAT* was detected in mouse, but not human, liver samples.

### GAN DIO-NASH mice demonstrate impaired glucose tolerance

Compared to chow-fed controls, GAN DIO-NASH mice showed significantly increased body weight, increased whole-body fat mass (adiposity) and hepatomegaly at termination (Fig. 4A-4D). An ipGTT was performed in chow-fed and GAN DIO-NASH mice one week before termination. While baseline and peak glucose levels were similar in chow-fed and GAN DIO-NASH mice, glucose excursions (t = 60–120 min, *p* = 0.0029–0.0002) and glucose-AUC levels (*p* = 0.0062) were significantly elevated in GAN-DIO NASH mice, signifying impaired glucose tolerance in GAN DIO-NASH mice (Fig. 4e). GAN DIO-NASH mice demonstrated hyperinsulinemia and correspondingly significantly increased HOMA-IR index compared to chow-fed mice (Fig. 4f, g). GAN-DIO NASH mice also exhibited hyperleptinemia and hypercholesterolemia (Fig. 4h, i). While plasma free fatty acid levels tended to be elevated (*p* = 0.05), plasma TG concentrations were unaltered (*p* = 0.75) in GAN DIO-NASH mice (Fig. 4j, k).

### Plasma and liver biochemical changes in GAN DIO-NASH mice

Other plasma and liver biochemical parameters analyzed are indicated in Table 3. GAN DIO-NASH mice showed significantly elevated levels of plasma markers of liver injury (ALT, AST; both *p* < 0.001) and fibrosis (total CK18, MMP-9, TIMP-1; all *p* < 0.0001). Compared to chow-fed mice, GAN DIO-NASH showed significantly increased concentrations of liver TG, TC and HP (all p < 0.0001). Elevated liver MMP-9 levels in GAN DIO-NASH mice did not attain statistical significance (*p* = 0.09).

**Table 3:** Elevated plasma and liver biochemical markers in GAN DIO-NASH mice

	Chow-fed C57BL/6 J mice	GAN DIO-NASH mice
Plasma ALT (U/L)	29.5 ± 1.9	166 ± 16***
Plasma AST (U/L)	65.4 ± 3.8	196 ± 16.8***
Plasma total CK-18 (ng/ml)	0.11 ± 0.02	0.83 ± 0.12***
Plasma MMP-9 (pg/ml)	15,409 ± 554	21,445 ± 990***
Plasma TIMP-1 (pg/ml)	1151 ± 56	4099 ± 453***
Liver TG (mg/g tissue)	7.4 ± 0.7	94.0 ± 5.4***
Liver TC (mg/g tissue)	2.0 ± 0.1	14.1 ± 0.6***
Liver HP (μg/mg tissue)	0.03 ± 0.01	0.08 ± 0.01***
Liver MMP-9 (pg/g tissue)	183,196 ± 7725	328,529 ± 80,679

*Abbreviations*: *ALT* alanine aminotransferase, *AST* aspartate aminotransferase, *CK-18* cytokeratin-18, *FFA* free fatty acids, *HP* hydroxyproline, *MMP-9* matrix metalloproteinase-9, *TC* total cholesterol, *TG* triglycerides; *TIMP-1* tissue inhibitor of metalloproteinase-1.

***p < 0.001, unpaired t-test (chow-fed mice, *n* = 10; GAN DIO-NASH mice, *n* = 15)

### Elevated levels of circulating and hepatic cytokines/chemokines in GAN DIO-NASH mice

Plasma and liver cytokine/chemokine levels are shown in Table 4. Both plasma and liver concentrations of TNF-α (plasma, p < 0.0001; liver, *p* = 0.0006), KC/GRO (CXCL1; plasma, p < 0.0001; liver, *p* = 0.0098) and MCP-1 (CCL2; plasma, *p* = 0.0014; liver, p < 0.0001) were significantly elevated in GAN DIO-NASH mice compared to chow-fed controls. In GAN DIO-NASH mice, most pronounced regulations were observed for hepatic MCP-1 expression. Also, GAN DIO-NASH mice displayed significantly elevated plasma IL-6 (*p* = 0.024) and IL-10 (*p* = 0.018) as well as liver IL-1β (*p* = 0.019) and IL-5 (*p* = 0.012) concentrations.

**Table 4:** Elevated plasma and liver cytokine levels in GAN DIO-NASH mice

Cytokine/ chemokine	Chow-fed C57BL/6 J mice		GAN DIO-NASH mice	
	Plasma (pg/ml)	Liver (pg/g tissue)	Plasma (pg/ml)	Liver (pg/g tissue)
TNF-α	7.4 ± 0.4	33 ± 2.8	15 ± 1.1***	78 ± 9.7***
IL-1β	*n.d.*	349 ± 46	*n.d.*	591 ± 79*
IL-2	*n.d.*	*n.d.*	*n.d.*	*n.d.*
IL-4	*n.d.*	*n.d.*	*n.d.*	*n.d.*
IL-5	1.9 ± 0.3	9.5 ± 0.7	2.0 ± 0.3	12.4 ± 0.7*
IL-6	8.2 ± 1.0	507 ± 19.7	53.7 ± 18.0*	478 ± 30
IL-10	14 ± 0.5	91 ± 7.2	18 ± 1.6*	97 ± 7.1
IL12p70	*n.d.*	530 ± 79	*n.d.*	658 ± 44
INF-γ	*n.d.*	9.9 ± 1.1	*n.d.*	9.0 ± 1.9
KC/GRO	58 ± 7.4	352 ± 32	138 ± 9.8***	640 ± 91**
MCP-1	11 ± 0.8	171 ± 9.3	30 ± 4.7**	1759 ± 304***

Plasma and liver cytokine levels in GAN DIO-NASH mice. *n.d.,* not detected (below lower level of quantification; IL-1β, 0.42 pg/ml; IL-2, 0.66 pg/ml; IL-4, 0.42 pg/ml; IL12p70, 7.69 pg/ml; INF-γ, 0.23 pg/ml). *p < 0.05, **p < 0.01, ***p < 0.001, unpaired t-test (chow-fed mice, *n* = 8; GAN DIO-NASH mice, n = 8)

## Discussion

Given the lack of evidence-based effective therapies for NASH, there is a marked need for animal models that better recapitulate cardinal features of the disease. The present study therefore assessed the clinical translatability of the GAN DIO-NASH mouse model by performing a head-to-head comparison of histological and transcriptome changes in liver biopsies from GAN DIO-NASH mice and human NASH patients. The main findings were significant clinical translatability of the GAN DIO-NASH mouse model with respect to the histopathological, transcriptional and metabolic aspects of human NASH. This highlights the suitability of the GAN DIO-NASH mouse model for identifying therapeutic targets and characterizing novel drug therapies for NASH.

Because liver diagnostic biopsy remains the definite criterion for confirming and grading of NASH, we compared liver biopsy histology in GAN DIO-NASH mice and NASH patients using the NAFLD activity scoring (NAS) and fibrosis staging system which is the most prevalent tool for defining NASH and assess histological activity [27]. GAN DIO-NASH mice showed similar pattern of liver lesions compared to the human validation set. As also seen in the clinic, GAN DIO-NASH mice displayed variation in disease severity which underscores the relevance of implementing liver biopsy-confirmed histopathology to control for within-subject disease progression and drug treatment efficacy in preclinical NASH models [7]. Histopathological scoring in GAN DIO-NASH mice recapitulated clinical criteria for diagnosing NASH. Pan-lobular hepatocyte lipid accumulation appeared mainly as macrovesicular steatosis in both GAN DIO-NASH mice and NASH patients. Lobular inflammation, ranging from mild to severe, in GAN DIO-NASH mice was characterized by foci consisting mainly of immune cell aggregates with macrophage and lymphocyte morphology which resembled human histopathology. Hepatocellular injury in GAN DIO-NASH mouse fulfilled criteria for mild hepatocyte ballooning degeneration [27], as indicated by the presence of few enlarged hepatocytes with vacuolated, rarified, pale cytoplasm. The rate and severity of ballooning injury in GAN DIO-NASH mice is similar to other high-fat diet-induced mouse models of NASH [7, 28, 40]. GAN DIO-NASH mice demonstrated liver fibrosis zonation patterns comparable to NASH patients. The majority of GAN DIO-NASH mice demonstrated mild or moderate fibrotic lesions. In addition to histological confirmation of disease hallmarks, GAN DIO-NASH mice reproducibly showed increased levels of plasma biomarkers known to be relevant to NAFLD/NASH diagnosis and assessment of fibrosis risk [41–43], including markers of hepatocellular injury (transaminases, CK-18) and ECM remodeling (MMP-9, TIMP-1). Enhanced ECM remodeling was also supported by liver biochemistry (HP, MMP-9).

As NAS and fibrosis scores are semiquantitative and provide a limited range of response data, imaging-based histomorphometry was applied for unbiased quantitative assessment of histological changes. The quantitative histological analysis confirmed marked lipid accumulation, increased immune cell infiltration and significant collagen deposition in GAN DIO-NASH mice and NASH patients. Consistent with the generally higher histopathological scores in the GAN DIO-NASH cohort compared to the NASH patients, GAN DIO-NASH mice showed relatively greater quantitative changes in histopathological hallmarks of NASH.

Hepatic transcriptome signatures in NASH patients and GAN DIO-NASH mice were compared and revealed similar dynamics in key gene expression pathways involved in NASH pathogenesis. As for histological changes, more profound changes in disease-relevant gene expression patterns were detected in GAN-DIO NASH mice. Commonalities in pathway perturbations suggested impaired carbohydrate and lipid metabolism. Consistent with the role of bile acids in NAFLD/NASH [44], NASH patients and GAN DIO-NASH mice showed gene expression signatures pointing to modulation of bile acid signaling. Chronic hepatic inflammation represents a major driver for development of NASH and is considered one of the strongest independent predictors for progression into fibrosis [45]. In agreement with aberrant innate and adaptive immune responses in NASH pathology [46, 47], GAN DIO-NASH mice showed gene regulations within both immune systems. Gene markers for monocyte recruitment, migration and activation were most consistently upregulated in GAN DIO-NASH mice, suggesting that increased abundance of pro-inflammatory macrophages play an important role in this model. In addition to activation of genes linked to programmed cell death, NASH patients and GAN DIO-NASH mice also demonstrated upregulated signaling pathways involved in cell cycle control which could suggest recruitment of signaling mechanisms related to cell division/carcinogenesis. Notably, the gene enrichment analysis also identified comprehensive changes in several ECM genes regulating hepatic collagen formation and turnover, supporting biochemical and histological evidence of extensive ECM remodeling activity in GAN DIO NASH mice and NASH patients. To obtain further resolution of hepatic transcriptome regulations in GAN DIO-NASH mice and NASH patients, RNA sequencing data were probed for genes encoding various protein targets exploited for the treatment of NASH, including regulators of hepatic lipid handling, adipogenesis, peripheral insulin sensitivity, immune cell function and ECM formation [7, 31, 32]. Compared to NASH patients, GAN DIO-NASH mice showed more widespread regulations within the defined gene set. Similar directional gene regulations in NASH patients and GAN DIO-NASH mice were associated with fatty acid and triglyceride synthesis (*ACC, DGAT1*), thyroid hormone receptor function (*THRB*), immune cell activation (*CCR5, LGALS3*) and collagen maturation (*SERPINH1*).

Corresponding to the clinical features of NASH [48], GAN DIO-NASH mice develop characteristics of the metabolic syndrome indicated by obesity, hypercholesterolemia and hyperinsulinemia with impaired glucose tolerance, the latter being a central feature of insulin resistance [49]. Similar to NASH patients, impaired insulin sensitivity in GAN DIO-NASH mice was supported by increased HOMA-IR, a widely used index for the estimation of insulin resistance [26]. By augmenting delivery of free fatty acids to the liver and increasing hepatic lipogenesis, insulin resistance is recognized as an important contributing mechanism for intrahepatic lipid accumulation and resulting lipotoxicity in NAFLD/NASH [50, 51]. As GAN DIO-NASH mice demonstrated profound hyperinsulinemia with normal baseline glucose levels this points to sustained pancreatic β cell compensation in this model. A similar insulin-resistant phenotype has also been reported in leptin-deficient *ob/ob* mice fed the GAN diet (GAN *ob/ob*-NASH mice) [22], an obesity-prone accelerated model of NASH, which lends further support to the translatability of GAN diet-based mouse models. While the GAN and AMLN diets induce a similar overall dysmetabolic phenotype in C57BL/6 J and *ob/ob* mice, AMLN diet feeding does not lead to manifest glucose intolerance [12–14, 22, 28]. This phenotypic difference has been ascribed to the more adipogenic properties of the GAN diet [22]. Similar to other high-fat/cholesterol diet-induced NAFLD/NASH models in C57BL/6 mice [5], GAN diet feeding did not promote hypertriglyceridemia. This is likely attributed to deficient hepatic triglyceride secretion as high-cholesterol diet feeding regimens have been reported to downregulate endogenous cholesterol ester and lipoprotein synthesis [52, 53]. In support of this, GAN DIO-NASH mice showed marked liver triglyceride and cholesterol accumulation together with reduced expression of several hepatic genes involved in cholesterol synthesis and transport. Collectively, development of severe steatosis in GAN DIO-NASH mice is likely a combined result of high-caloric diet intake, impaired liver lipid catabolism and reduced lipid clearance.

## Conclusions

In conclusion, the GAN DIO-NASH mouse demonstrates good clinical translatability with respect to the physiological, metabolic and histopathological aspects of fibrosing NASH along with a strong concordance with the liver transcriptome signature of the human disease. Hence, the clear metabolic and histopathological hallmarks of NASH highlight the suitability of the GAN DIO-NASH mouse model for identifying therapeutic targets and characterizing novel drug therapies for NASH.

## Supplementary information

**Additional file 1: Table S1.** In-house gene panel on candidate genes associated with NASH and fibrosis.

## Data Availability

RNA sequencing data are accessible at the NCBI GEO database under accession no. GSE126848. Other datasets used and/or analyzed during the current study are available from the corresponding author on reasonable request.

## Abbreviations

ALT: Alanine aminotransferase
AST: Aspartate aminotransferase
AMLN: Amylin liver NASH
CK-18: Cytokeratin 18
DIO: Diet-induced obesity
GAN: Gubra Amylin NASH
HP: Hydroxyproline
ipGTT: Intraperitoneal glucose tolerance test
MMP-9: Matrix metalloproteinase-9
NAFLD: Non-alcoholic fatty liver disease
NAS: NAFLD Activity Score
NASH: Non-alcoholic steatohepatitis
PCA: Principal component analysis
PSR: Picro-Sirius Red
TC: Total cholesterol
TG: Triglycerides
TIMP-1: Tissue inhibitor of metalloproteinase-1
