# Supplementary material for: Human translatability of the GAN diet-induced obese mouse model of non-alcoholic steatohepatitis
Source: BMC Gastroenterol. 2020 Jul 6;20:210. doi: 10.1186/s12876-020-01356-2 (PMC7336447; doi:10.1186/s12876-020-01356-2)
Supplement: Supplementary file 1 — Additional file 1: Table S1. In-house gene panel on candidate genes associated with NASH and fibrosis. [file 12876_2020_1356_MOESM1_ESM.docx]

| **Accession key**  **Table S1. In-house gene panel on candidate genes associated with NASH and fibrosis** | **Gene name** | **Gene set** | **Description** |
| --- | --- | --- | --- |
| ENSG00000005471 | ABCB4 (MDR2/3) | Bile acid metabolism | ATP binding cassette subfamily B member 4 |
| ENSG00000073734 | ABCB11 (BSEP) | Bile acid metabolism | ATP binding cassette, subfamily B member 11 |
| ENSG00000278540 | ACACA (ACC1) | Lipid metabolism | acetyl-CoA carboxylase alpha |
| ENSG00000076555 | ACACB (ACC2) | Lipid metabolism | acetyl-CoA carboxylase beta |
| ENSG00000107796 | ACTA2 (A-SMA) | ECM organization | actin, alpha 2, smooth muscle, aorta |
| ENSG00000174837 | ADGRE1 (F4/80, EMR1) | Inflammation | adhesion G protein-coupled receptor E1 |
| ENSG00000163568 | AIM2 | Hepatocellular cell death | absent in melanoma 2 |
| ENSG00000142208 | AKT1 | Glucose metabolism | AKT serine/threonine kinase 1 |
| ENSG00000128272 | ATF4 | ER stress | activating transcription factor 4 |
| ENSG00000118217 | ATF6 | ER stress | activating transcription factor 6 |
| ENSG00000131471 | AOC3 (VAP-1) | Inflammation | amine oxidase copper containing 3 |
| ENSG00000118137 | APOA1 | Lipid metabolism | apolipoprotein A1 |
| ENSG00000110243 | APOA5 | Lipid metabolism | apolipoprotein A5 |
| ENSG00000234906 | APOC2 | Lipid metabolism | apolipoprotein C2 |
| ENSG00000110245 | APOC3 | Lipid metabolism | apolipoprotein C3 |
| ENSG00000137752 | CASP1(BACS) | Hepatocellular cell death | caspase 1 |
| ENSG00000164305 | CASP3 | Hepatocellular cell death | caspase 3 |
| ENSG00000138794 | CASP6 | Hepatocellular cell death | caspase 6 |
| ENSG00000165806 | CASP7 | Hepatocellular cell death | caspase 7 |
| ENSG00000064012 | CASP8 | Hepatocellular cell death | caspase 8 |
| ENSG00000213341 | CHUK (IKK) | Inflammation | conserved helix-loop-helix ubiquitous kinase |
| ENSG00000108691 | CCL2 (MCP-1) | Inflammation | C-C motif chemokine ligand 2 |
| ENSG00000271503 | CCL5 (RANTES) | Inflammation | C-C motif chemokine ligand 5 |
| ENSG00000115009 | CCL20 (MIP3A) | Inflammation | C-C motif chemokine ligand 20 |
| ENSG00000163823 | CCR1 | Inflammation | C-C motif chemokine receptor 1 |
| ENSG00000121807 | CCR2 | Inflammation | C-C motif chemokine receptor 2 |
| ENSG00000160791 | CCR5 | Inflammation | C-C motif chemokine receptor 5 |
| ENSG00000170458 | CD14 | Inflammation | CD14 molecule |
| ENSG00000135218 | CD36 | Lipid metabolism | CD36 molecule |
| ENSG00000129226 | CD68 | Inflammation | CD68 molecule |
| ENSG00000114013 | CD86 | Inflammation | CD86 molecule |
| ENSG00000177575 | CD163 | Inflammation | CD163 molecule |
| ENSG00000108821 | COL1A1 | ECM organization | collagen type I alpha 1 chain |
| ENSG00000164692 | COL1A2 | ECM organization | collagen type I alpha 2 chain |
| ENSG00000168542 | COL3A1 | ECM organization | collagen type III alpha 1 chain |
| ENSG00000187498 | COL4A1 | ECM organization | collagen type IV alpha 1 chain |
| ENSG00000130635 | COL5A1 | ECM organization | collagen type V alpha 1 chain |
| ENSG00000204262 | COL5A2 | ECM organization | collagen type V alpha 2 chain |
| ENSG00000080573 | COL5A3 | ECM organization | collagen type V alpha 3 chain |
| ENSG00000142156 | COL6A1 | ECM organization | collagen type VI alpha 1 chain |
| ENSG00000142173 | COL6A2 | ECM organization | collagen type VI alpha 2 chain |
| ENSG00000163359 | COL6A3 | ECM organization | collagen type VI alpha 3 chain |
| ENSG00000084636 | COL16A1 | ECM organization | collagen type 16 alpha 1 chain |
| ENSG00000110090 | CPT1A/CPT1 | Lipid metabolism | carnitine palmitoyltransferase 1A |
| ENSG00000088882 | CPXM1 | ECM organization | carboxypeptidase X, M14 family member 1 |
| ENSG00000143387 | CTSK | ECM organization | cathepsin K |
| ENSG00000167910 | CYP7A1 | Bile acid metabolism | cytochrome P450 family 7 subfamily A member 1 |
| ENSG00000180432 | CYP8B1 | Bile acid metabolism | cytochrome P450 family 8 subfamily B member 1 |
| ENSG00000173198 | CYSLTR1 | Inflammation | cysteinyl leukotriene receptor 1 |
| ENSG00000175197 | DDIT3 (CHOP) | Hepatocellular cell death | DNA damage-inducible transcript 3 |
| ENSG00000185000 | DGAT1 | Lipid metabolism | diacylglycerol O-acyltransferase 1 |
| ENSG00000115380 | EFEMP1 | ECM organization | EGF-containing fibulin-like extracellular matrix protein 1 |
| ENSG00000172071 | EIF2AK3 (PERK) | ER stress | eukaryotic translation initiation factor 2-alpha kinase 3 |
| ENSG00000126767 | ELK1 | Glucose metabolism | ELK1, ETS transcription factor |
| ENSG00000178607 | ERN1 (IRE1) | ER stress | endoplasmic reticulum to nucleus signaling 1 |
| ENSG00000169710 | FASN | Lipid metabolism | fatty acid synthase |
| ENSG00000140092 | FBLN5 (DANCE) | ECM organization | fibulin-5 |
| ENSG00000162344 | FGF19 | Bile acid metabolism | fibroblast growth factor 19 |
| ENSG00000105550 | FGF21 | Bile acid metabolism | fibroblast growth factor 21 |
| ENSG00000170345 | FOS | Inflammation | Fos proto-oncogene, AP-1 transcription factor subunit |
| ENSG00000150907 | FOXO1 | Glucose metabolism | forkhead box O1 |
| ENSG00000131482 | G6PC (G6PASE) | Glucose metabolism | glucose-6-phosphatase catalytic subunit |
| ENSG00000112164 | GLP1R | Glucose metabolism | glucagon like peptide 1 receptor |
| ENSG00000158669 | GPAT4 | Lipid metabolism | glycerol-3-phosphate acyltransferase 4 |
| ENSG00000179921 | GPBAR1 (TGR5) | Bile acid metabolism | G protein-coupled bile acid receptor 1 |
| ENSG00000177885 | GRB2 | Glucose metabolism | growth factor receptor bound protein 2 |
| ENSG00000111713 | GYS2 | Glucose metabolism | glycogen synthase 2 |
| ENSG00000113161 | HMGCR | Lipid metabolism | 3-hydroxy-3-methylglutaryl-CoA reductase |
| ENSG00000112972 | HMGCS1 | Lipid metabolism | 3-hydroxy-3-methylglutaryl-CoA synthase 1 |
| ENSG00000134240 | HMGCS2 | Lipid metabolism | 3-hydroxy-3-methylglutaryl-CoA synthase 2 |
| ENSG00000125538 | IL1B | Inflammation | interleukin 1 beta |
| ENSG00000150782 | IL18 | Hepatocellular cell death | interleukin 18 |
| ENSG00000171105 | INSR | Glucose metabolism | insulin receptor |
| ENSG00000169047 | IRS1 | Glucose metabolism | insulin receptor substrate 1 |
| ENSG00000177606 | JUN | Inflammation | Jun proto-oncogene, AP-1 transcription factor subunit |
| ENSG00000138030 | KHK | Glucose metabolism | ketohexokinase |
| ENSG00000050555 | LAMC3 | ECM organization | laminin subunit gamma-3 |
| ENSG00000130164 | LDLR | Lipid metabolism | low density lipoprotein receptor |
| ENSG00000131981 | LGALS3 (MAC-2) | Inflammation | galectin 3 |
| ENSG00000134013 | LOXL2 | ECM organization | lysyl oxidase like 2 |
| ENSG00000138131 | LOXL4 | ECM organization | lysyl oxidase like 4 |
| ENSG00000123384 | LRP1 | Lipid metabolism | LDL receptor related protein 1 |
| ENSG00000119681 | LTBP2 | ECM organization | Latent Transforming Growth Factor Beta Binding Protein 2 |
| ENSG00000100030 | MAPK1 | Glucose metabolism | mitogen-activated protein kinase 1 |
| ENSG00000197442 | MAP3K5 (ASK-1) | Inflammation | mitogen-activated protein kinase kinase kinase 5 |
| ENSG00000107643 | MAPK8 (JNK) | Inflammation | mitogen-activated protein kinase 8 |
| ENSG00000076706 | MCAM (CD146) | ECM organization | melanoma cell adhesion molecule |
| ENSG00000169032 | MAP2K1 (MEK1) | Glucose metabolism | mitogen-activated protein kinase kinase 1 |
| ENSG00000126934 | MAP2K2 (MEK2) | Glucose metabolism | mitogen-activated protein kinase kinase 2 |
| ENSG00000087245 | MMP2 | ECM organization | matrix metallopeptidase 2 |
| ENSG00000100985 | MMP9 | ECM organization | matrix metallopeptidase 9 |
| ENSG00000125966 | MMP24 | ECM organization | matrix metallopeptidase 24 |
| ENSG00000011028 | MRC2 | ECM organization | mannose receptor C type 2 |
| ENSG00000198793 | MTOR | Glucose metabolism | mechanistic target of rapamycin kinase |
| ENSG00000109320 | NFKB1 (NF-KB) | Inflammation | nuclear factor kappa B subunit 1 |
| ENSG00000131910 | NR0B2 (SHP) | Bile acid metabolism | nuclear receptor subfamily 0 group B member 2 |
| ENSG00000091106 | NLRC4 (IPAF) | Hepatocellular cell death | NLR family CARD domain containing 4 |
| ENSG00000091592 | NLRP1 (NLRP1B) | Hepatocellular cell death | NLR family pyrin domain containing 1 |
| ENSG00000162711 | NLRP3 | Hepatocellular cell death | NLR family pyrin domain containing 3 |
| ENSG00000025434 | NR1H3 (LXR-α) | Inflammation | nuclear receptor subfamily 1 group H member 3 |
| ENSG00000012504 | NR1H4 (FXR) | Lipid metabolism | nuclear receptor subfamily 1 group H member 4 |
| ENSG00000185386 | MAPK11 (P38) | Inflammation | mitogen-activated protein kinase 11 |
| ENSG00000197461 | PDGFA (PDGF) | ECM organization | platelet derived growth factor subunit A |
| ENSG00000186951 | PPARA | Lipid metabolism | peroxisome proliferator activated receptor alpha |
| ENSG00000112033 | PPARD | Lipid metabolism | peroxisome proliferator activated receptor delta |
| ENSG00000132170 | PPARG | Lipid metabolism | peroxisome proliferator activated receptor gamma |
| ENSG00000124253 | PCK1 (PEPCK) | Glucose metabolism | phosphoenolpyruvate carboxykinase 1 |
| ENSG00000163558 | PRKCI (PKC) | Glucose metabolism | protein kinase C iota |
| ENSG00000067606 | PRKCZ (PKC) | Glucose metabolism | protein kinase C zeta |
| ENSG00000100504 | PYGL (PYG) | Glucose metabolism | phosphorylase, glycogen, liver |
| ENSG00000137275 | RIPK1 (RIP1) | Hepatocellular cell death | receptor interacting serine/threonine kinase 1 |
| ENSG00000129465 | RIPK3 (RIP3) | Hepatocellular cell death | receptor interacting serine/threonine kinase 3 |
| ENSG00000073060 | SCARB1 | Lipid metabolism | scavenger receptor class B member 1 |
| ENSG00000099194 | SCD (SCD1) | Lipid metabolism | stearoyl-CoA desaturase |
| ENSG00000149257 | SERPINH1 (HSP47) | ECM organization | serpin family H member 1 |
| ENSG00000181856 | SLC2A4 (GLUT4) | Glucose metabolism | solute carrier family 2 member 4 |
| ENSG00000125255 | SLC10A2 (IBAT) | Bile acid metabolism | solute carrier family 10 member 2 |
| ENSG00000137204 | SLC22A7 (OAT2) | Bile acid metabolism | solute carrier family 22 member 7 |
| ENSG00000083807 | SLC27A5 | Bile acid metabolism | solute carrier family 27 member 5 |
| ENSG00000186198 | SLC51B (OSTB) | Bile acid metabolism | solute carrier family 51 beta subunit |
| ENSG00000084453 | SLCO1A2 | Bile acid metabolism | solute carrier organic anion transporter family member 1A2 |
| ENSG00000175387 | SMAD2 | Inflammation signaling | SMAD family member 2 |
| ENSG00000166949 | SMAD3 | Inflammation signaling | SMAD family member 3 |
| ENSG00000141646 | SMAD4 | Inflammation signaling | SMAD family member 4 |
| ENSG00000104549 | SQLE | Lipid metabolism | squalene epoxidase |
| ENSG00000072310 | SREBF1 | Glucose metabolism | sterol regulatory element binding transcription factor 1 |
| ENSG00000105329 | TGFB1 (TGFB) | ECM organization | transforming growth factor beta 1 |
| ENSG00000106799 | TGFBR1 (TGFBR) | Inflammation | transforming growth factor beta receptor 1 |
| ENSG00000151090 | THRB | Lipid metabolism | thyroid hormone receptor beta |
| ENSG00000102265 | TIMP1 | ECM organization | TIMP metallopeptidase inhibitor 1 |
| ENSG00000035862 | TIMP2 | ECM organization | TIMP metallopeptidase inhibitor 2 |
| ENSG00000100234 | TIMP3 | ECM organization | TIMP metallopeptidase inhibitor 3 |
| ENSG00000136869 | TLR4 | Inflammation | toll like receptor 4 |
| ENSG00000232810 | TNF (TNFA) | Inflammation | tumor necrosis factor |
| ENSG00000067182 | TNFRSF1A (TNFR) | Inflammation | TNF receptor superfamily member 1A |
| ENSG00000127191 | TRAF2 | Inflammation | TNF receptor associated factor 2 |
| ENSG00000175104 | TRAF6 | Inflammation | TNF receptor associated factor 6 |
| ENSG00000038427 | VCAN | ECM organization | versican |
| ENSG00000147852 | VLDLR | Lipid metabolism | very low density lipoprotein receptor |
| ENSG00000100219 | XBP1 | ER stress | X-box binding protein 1 |

**Supplementary Table 1.**
